# A bioorthogonal chemical reporter for fatty acid synthase–dependent protein acylation

**DOI:** 10.1016/j.jbc.2021.101272

**Published:** 2021-10-01

**Authors:** Krithika P. Karthigeyan, Lizhi Zhang, David R. Loiselle, Timothy A.J. Haystead, Menakshi Bhat, Jacob S. Yount, Jesse J. Kwiek

**Affiliations:** 1 Department of Microbiology and Center for Retrovirus Research, The Ohio State University, Columbus, Ohio, USA; 2 Department of Microbial Infection and Immunity, The Ohio State University, Columbus, Ohio, USA; 3 Department of Pharmacology and Cancer Biology, Duke University School of Medicine, Durham, North Carolina, USA

**Keywords:** fatty acid synthase, click chemistry, S-palmitoylation, N-myristoylation, HIV-1, influenza, fatty acylation, IFITM3, HIV-1 Gag, CD9, Alk-3, 4-pentynoic acid, Alk-4, alkynyl acetic acid or 5-hexynoic acid, Alk-12, alkynyl myristic acid or 13-tetradecynoic acid, Alk-16, alkynyl palmitic acid, or 15-hexadecynoic acid, CTNB1, catenin beta-1, DHHC, aspartate–histidine–histidine–cysteine, DMSO, dimethyl sulfoxide, FA, fatty acid, FASN, fatty acid synthase, FBS, fetal bovine serum, GNA1I, guanine nucleotide-binding protein, alpha inhibiting 1, HA, hemagglutinin, IFITM3, interferon-induced transmembrane protein 3, IFNβ, interferon beta, KS6A1, ribosomal protein S6 kinase alpha, MA, matrix, SAMHD1, SAM domain and HD domain containing protein 1

## Abstract

Mammalian cells acquire fatty acids (FAs) from dietary sources or *via de novo* palmitate production by fatty acid synthase (FASN). Although most cells express FASN at low levels, it is upregulated in cancers of the breast, prostate, and liver, among others, and is required during the replication of many viruses, such as dengue virus, hepatitis C, HIV-1, hepatitis B, and severe acute respiratory syndrome coronavirus 2, among others. The precise role of FASN in disease pathogenesis is poorly understood, and whether *de novo* FA synthesis contributes to host or viral protein acylation has been traditionally difficult to study. Here, we describe a cell-permeable and click chemistry–compatible alkynyl acetate analog (alkynyl acetic acid or 5-hexynoic acid [Alk-4]) that functions as a reporter of FASN-dependent protein acylation. In an FASN-dependent manner, Alk-4 selectively labels the cellular protein interferon-induced transmembrane protein 3 at its known palmitoylation sites, a process that is essential for the antiviral activity of the protein, and the HIV-1 matrix protein at its known myristoylation site, a process that is required for membrane targeting and particle assembly. Alk-4 metabolic labeling also enabled biotin-based purification and identification of more than 200 FASN-dependent acylated cellular proteins. Thus, Alk-4 is a useful bioorthogonal tool to selectively probe FASN-mediated protein acylation in normal and diseased states.

Long-chain fatty acids (FAs) are essential components of lipid bilayers, are used to store energy liberated by β-oxidation, and are covalently attached to proteins to increase hydrophobicity and regulate subcellular localization (1). In mammalian cells, long-chain FAs can be obtained exogenously through dietary sources or endogenously *via de novo* FA biosynthesis (2). Mammalian fatty acid synthase (FASN) is a 272 kDa cytosolic enzyme that catalyzes the complete *de novo* synthesis of palmitate from acetyl-CoA and malonyl-CoA. The final product, palmitic acid (16:0), is then released from the thioesterase domain of FASN and can then be metabolized by β-oxidation into myristic acid in the mitochondria (14:0), elongated to other long-chain FA such as stearic acid, or elongated and desaturated into oleic acid in the endoplasmic reticulum (3). This process is aided by acyl-CoA synthetases, which activate the free FAs produced by FASN to their coenzyme-A linked thioesters. FAs synthesized *de novo* or acquired through dietary sources can be covalently attached to proteins by acyltransferases such as palmitoyl transferases and N-myristoyl transferases in a process called fatty acylation (1). FASN expression is highly regulated in cells, and its expression can change dramatically in response to stresses, such as starvation, lactation, or pathological states (3). Increased *de novo* FA biosynthesis and FASN upregulation have been observed in breast cancer, melanoma, and hepatocellular carcinoma (4). Studies of enveloped viruses including hepatitis B virus (5), dengue virus (6), Epstein–Barr virus (7), hepatitis C virus (8), HIV-1 (9), chikungunya virus (10, 11), and West Nile virus (12, 13) indicate that many viruses both upregulate and require host FASN activity for effective replication. The contributions of *de novo*–synthesized FA to post-translational modifications of viral and host proteins remain understudied.

Identification of protein acylation has been challenging because of the lack of antibodies against lipid modifications and inefficiencies of standard mass spectrometry techniques to identify acylated proteins (14). While protein myristoylation site prediction is facilitated by a consensus sequence motif on nearly all myristoylated proteins (Met-Gly-XXX-Ser/Thr) (1), protein palmitoylation site prediction remains challenging because of the lack of a consensus sequence (15). Measuring acyl-group synthesis mediated by FASN and the fate of the *de novo*–synthesized FAs using ^3^H labeled acetate suffers from low detection sensitivity, general complications associated with radioisotope work (16), and an inability to selectively enrich acylated proteins. Over the last decade, bioorthogonal labeling and detection of protein fatty acylation using click chemistry–compatible analogs of palmitate and myristate have provided quick and sensitive methods for detection of protein acylations (17, 18). The copper-catalyzed azide–alkyne cycloaddition reactions enable labeling of cells with alkynyl analogs of FAs that can be reacted with azides conjugated to suitable detection tags, such as fluorophores, or affinity tags, including biotin (19, 20). Although very useful, palmitate and myristate analogs only measure the acylation state of proteins modified by the exogenous chemical reporters. Given the critical role of FASN-dependent *de novo*–synthesized FAs in cancer, metabolic disorders, and viral replication, we posit that a bioorthogonal reporter of FASN-dependent protein acylation will facilitate a better understanding of the contributions of FASN-dependent protein fatty acylation to protein function, protein localization, and FASN-mediated pathogenesis. Here, we demonstrate the utility of alkynyl acetic acid or 5-hexynoic acid (Alk-4), a cell-permeable and click chemistry compound that labels proteins acylated by products of FASN-mediated *de novo* FA biosynthesis.

## Results

### Alk-4 labels palmitoylated proteins at known palmitoylation sites

**Figure 1:**
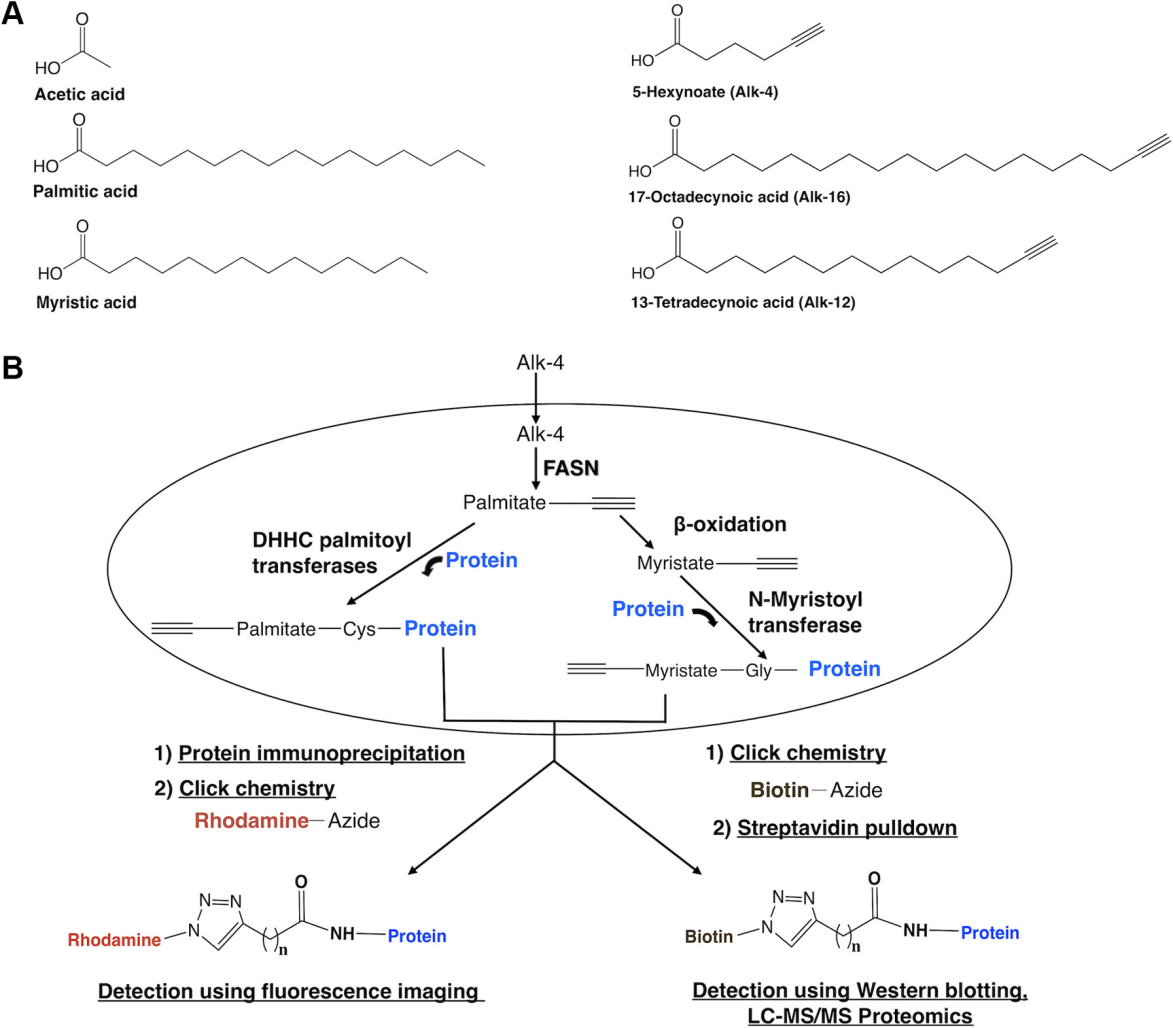
**Analog structures and schema depicting Alk-4 metabolism and incorporation onto protein acylation sites.***A*, structures of acetate, palmitate, and myristate, followed by their click chemistry–compatible analogs Alk-4, Alk-16, and Alk-12. *B*, 5-Hexynoic acid (Alk-4) can be metabolized through the endogenous FASN pathway to yield functionalized versions of fatty acyl groups that are transferred onto protein acylation sites by palmitoyl or N-myristoyl transferases. Copper-catalyzed azide–alkyne cycloaddition (CuAAC) click reaction of proteins containing the functionalized alkyne group to an azide-conjugated fluorophore such as rhodamine can be used for fluorescence imaging, whereas CuAAC click reaction of alkyne-containing proteins to an azide-conjugated biotin can be used for affinity purification and subsequent Western blotting or proteomics. FASN, fatty acid synthase.

**Figure 2:**
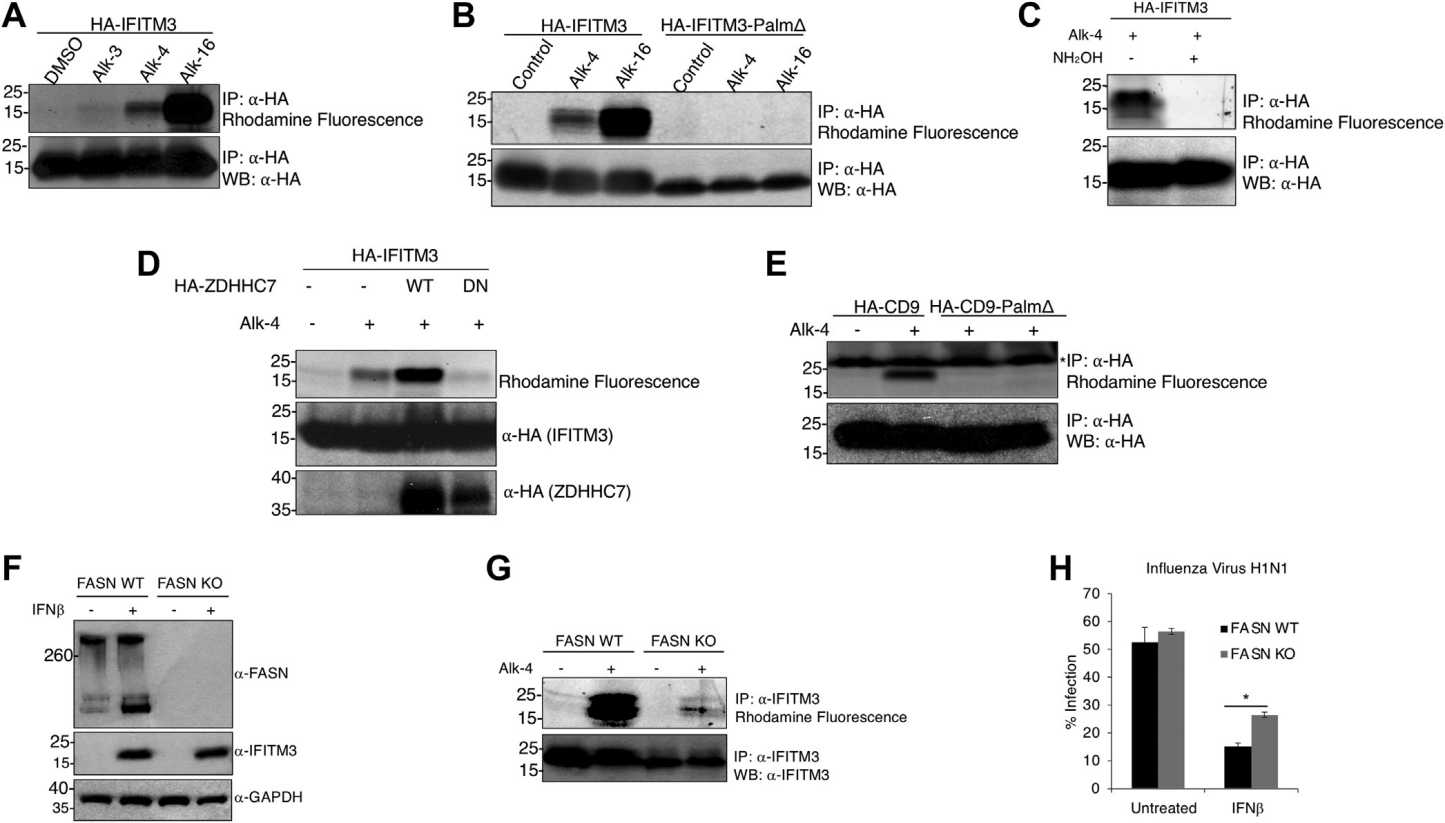
**FASN-dependent incorporation of Alk-4 at known protein palmitoylation sites.***A*, immunoprecipitated HA-IFITM3 from HA-IFITM3 transfected 293Ts followed by rhodamine azide click reaction revealed detectable labeling by Alk-16 and Alk-4 and minimal labeling by Alk-3. *B*, a triple cysteine to alanine IFITM3 mutant (PalmΔ) was not labeled by Alk-4, suggesting that Alk-4 labeling of IFITM3 occurred on known palmitoylated cysteines. *C*, Alk-4 labeling of IFITM3 is removed by hydroxylamine treatment, which removes palmitate groups from S-palmitoylated proteins. *D*, Alk-4 labeled CD9, whereas a mutant where its six palmitoylated cysteines were mutated to alanine was not labeled, revealing that Alk-4 labels CD9 on known palmitoylated cysteines (∗ indicates 25 kDa fluorescent molecular weight standard bleed through). *E*, DHHC palmitoyltransferase overexpression increased Alk-4 labeling of IFITM3, whereas a dominant negative mutant partially decreased labeling, suggesting that Alk-4 is metabolized into a long-chain fatty acid utilized by DHHC palmitoyltransferases. *F*, to test the requirement of FASN for labeling of endogenous IFITM3 in cells treated with IFNβ, HAP1 WT and FASN KO cells were labeled with Alk-4. Western blotting was done to confirm FASN levels in WT and KO cells and expression of endogenous IFITM3 on IFNβ treatment. *G*, Alk-4 labeling of endogenous IFITM3 was only observed in WT cells and not detected in FASN KO cells, indicating that FASN contributes to palmitoylation of IFITM3. *H*, IFNβ was significantly less effective at inhibiting influenza virus strain H1N1 infection in FASN KO cells (∗*p* = 0.0002), indicating that FASN is required for mounting of an effective IFNβ immune response against influenza virus, possibly through provision for fatty acyl groups for activation of IFITM3. DHHC, aspartate–histidine–histidine–cysteine; FASN, fatty acid synthase; HA, hemagglutinin; IFITM3, interferon-induced transmembrane protein 3; IFNβ, interferon beta.

Bioorthogonal reporters such as alkynyl palmitate (alkynyl palmitic acid or 15-hexadecynoic acid [Alk-16]) and alkynyl myristic acid or 13-tetradecynoic acid (Alk-12) are substrates of palmitoyltransferase and myristoyltransferase activity that are often used to identify palmitoylated and myristoylated proteins (21, 22, 23). Alk-12 and Alk-16 are exogenous FA analogs that mimic the end product of FASN activity (palmitate) or the β-oxidation product of palmitate (myristate) and thus cannot be used to determine if *de novo*–synthesized FAs can be incorporated onto protein acylation sites. We hypothesized that a cell-permeable and bioorthogonal mimic of a putative FASN substrate, 5-hexynoate (termed Alk-4 here) (24), could be used to study the contributions of FASN-mediated *de novo* FA synthesis to protein acylation (Fig. 1, *A* and *B*). To determine whether Alk-4 selectively labels palmitoylated proteins, we tested whether a known palmitoylated protein, interferon-induced transmembrane protein 3 (IFITM3), was labeled upon a 24-h treatment of cells with alkynyl acetate analogs of different carbon chain lengths, 4-pentynoic acid (Alk-3) and Alk-4, in comparison with the well-established palmitoylation reporter Alk-16 (Fig. 1, *A* and *B*). Alk-16 robustly labeled IFITM3 as detected by click chemistry tagging of immunoprecipitated IFITM3 with azidorhodamine and fluorescence gel scanning. Alk-4 also successfully labeled IFITM3, whereas Alk-3 showed minimal labeling (Fig. 2*A*). Next, we tested whether Alk-4 labeling of IFITM3 occurred on its known palmitoylated cysteines (22). A triple cysteine to alanine palmitoylation-deficient mutant of IFITM3 (termed PalmΔ) was not labeled by either Alk-16 or Alk-4 (Fig. 2*B*). Hydroxylamine treatment is known to cleave the palmitoyl–thioester linkage (25), and hydroxylamine treatment removed labeling of IFITM3 by Alk-4 (Fig. 2*C*). To further test the ability of Alk-4 to label palmitoylated proteins, we examined whether the tetraspanin CD9, which has six palmitoylated cysteines, was also labeled by Alk-4. Similar to IFITM3, CD9 was labeled by Alk-4, whereas a mutant CD9 in which its palmitoylated cysteines were mutated to alanine (termed CD9-PalmΔ) was not labeled (Fig. 2*D*). These results indicate that Alk-4 is metabolized into a click chemistry–functionalized FA adduct that is specifically incorporated onto protein palmitoylation sites.

### Alk-4 metabolism provides a substrate used by aspartate–histidine–histidine–cysteine palmitoyltransferases

Many proteins are reversibly palmitoylated at cysteine residues (26) by aspartate–histidine–histidine–cysteine (DHHC) palmitoyltransferases. DHHC palmitoyltransferases primarily use palmitoyl-CoA (C16:0) to modify cysteine residues on proteins, although DHHCs can tolerate substrates with carbon chain lengths as short as 14 and as long as 20 (27, 28). Acyl chains with fewer than 14 carbons have not been detected on cysteines, indicating that DHHC enzymes disfavor short-chain FAs as substrates (29, 30, 31, 32). Given the selectivity of the DHHC palmitoyltransferases for long-chain FAs, we sought to determine whether labeling of IFITM3 by Alk-4 was affected by DHHC7 overexpression, which was previously shown to be among the enzymes that can catalyze IFITM3 palmitoylation (33). In cells incubated with Alk-4, DHHC7 overexpression increased IFITM3 labeling, whereas overexpression of a dominant negative DHHC7 mutant decreased IFITM3 labeling (Fig. 2*E*). These results indicate that Alk-4 is metabolized into a long-chain FA that can be used as a substrate by DHHC palmitoyltransferases for protein palmitoylation.

### Labeling of IFITM3 by Alk-4 requires FASN

We have previously shown that IFITM3 palmitoylation is required for its antiviral activity against influenza virus infection (22, 34). To determine if FASN-mediated *de novo* FA biosynthesis contributes to an interferon beta (IFNβ)–regulated IFITM3-mediated antiviral response, we measured endogenous IFITM3 labeling by Alk-4 in WT and FASN KO HAP1 cells. As expected, IFNβ induced endogenous IFITM3 expression, and IFITM3 upregulation was independent of FASN expression (Fig. 2*F*). In WT cells, Alk-4 treatment resulted in robust endogenous IFITM3 labeling that was absent in FASN-deficient cells (Fig. 2*G*). Thus, we show for the first time that FASN contributes to the palmitoylation of endogenous IFITM3. Owing to the observations that IFITM3 is required for an effective IFNβ-mediated anti-influenza response (33), that palmitoylation of IFITM3 is required for its antiviral activity (33), and that FASN is required for Alk-4–mediated IFITM3 palmitoylation (Fig. 2*G*), we sought to determine the effect of FASN expression on IFNβ-mediated inhibition of influenza virus infection. In the absence of IFNβ, FASN expression had no effect on influenza infection (Fig. 2*H*). However, IFNβ-mediated inhibition of influenza virus infection was significantly decreased in the absence of FASN expression (Fig. 2*H*), suggesting that FASN-dependent palmitate synthesis likely contributes to the palmitoylation-dependent antiviral activity of IFITM3.

### Alk-4 labeling of myristoylated proteins is FASN dependent

**Figure 3:**
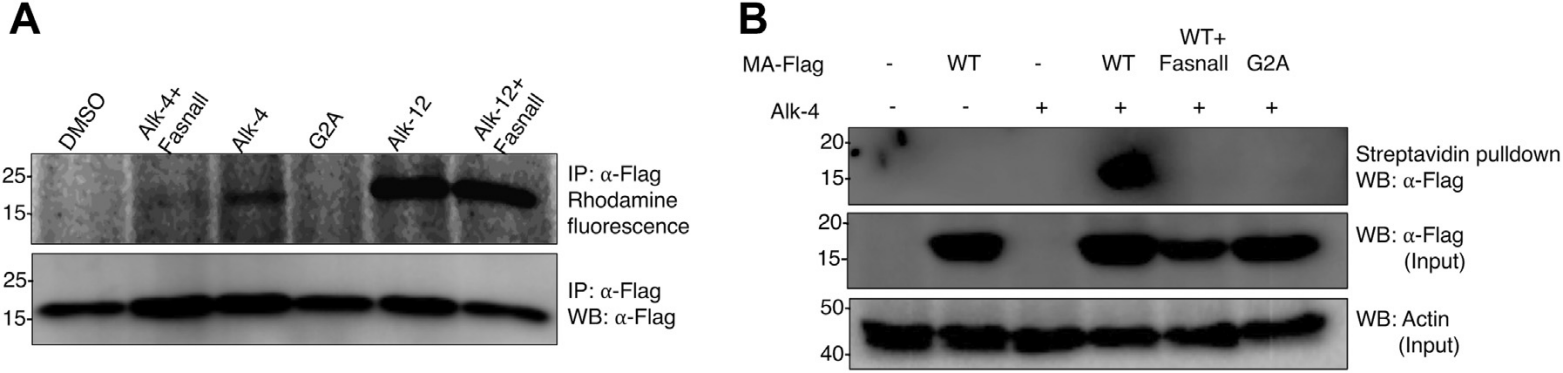
**FASN-dependent incorporation of Alk-4 at known myristoylation sites.** 293Ts were transfected overnight with plasmids encoding MA-Flag (MA) or the myristoylation-deficient G2A-MA-Flag (G2A) and incubated for 24 h either with Alk-4 or Alk-12. About 10 μM of the FASN inhibitor Fasnall was added 1 h post-transfection. *A*, immunoprecipitation of MA-Flag and click reaction with TAMRA azide revealed labeling of WT MA with Alk-4 and not G2A-MA, suggesting that Alk-4 labels MA at its known myristoylation site. FASN inhibition with Fasnall reduced labeling of MA by Alk-4, suggesting that FASN is required for labeling of MA by Alk-4. Alk-12 also labeled MA in both cells treated and untreated with Fasnall, indicating that Fasnall does not inhibit NMT function. *B*, on click reaction with diazo azidobiotin, streptavidin pulldown, and selective elution of Alk-4 labeled proteins, only WT-MA was labeled by Alk-4 and not G2A-MA, and Fasnall treatment also inhibited Alk-4 labeling of MA, corroborating our findings that FASN is required for labeling of MA by Alk-4 at its myristoylation site. FASN, fatty acid synthase.

Acetyl-CoA is condensed with malonyl-CoA and elongated by FASN to generate palmitate for protein palmitoylation. To generate myristoyl-CoA for myristoylation, palmitate is activated to palmitoyl-CoA by palmitoyl-CoA synthetase, which is then β-oxidized to myristoyl-CoA before it is covalently attached to glycine residues by N-myristoyl transferases (1, 35). To determine if Alk-4 is metabolized into an FA analog that can selectively label myristoylated proteins, we tested whether a known myristoylated protein, HIV-1 matrix (MA) protein, was labeled upon a 24-h incubation with Alk-4. Human embryonic kidney 293T cells were transfected with Flag-tagged HIV-1 MA or the myristoylation-deficient MA-G2A mutant (MA-G2A) and treated with Alk-4 or Alk-12 (an established chemical reporter of myristoylation). Immunoprecipitation of Flag-tagged MA and subsequent click reaction with azidorhodamine revealed labeling of HIV-1 MA in cells incubated with Alk-4 or Alk-12 (Fig. 3*A*). The G2A-MA Gag protein, which cannot be myristoylated, was not labeled by Alk-4, indicating myristoylation site–specific labeling of HIV-1 MA protein by Alk-4. Treatment of cells with Fasnall (9, 36), an FASN inhibitor, abolished Alk-4 labeling of HIV-1 MA protein. As a control, Fasnall treatment did not disrupt HIV-1 MA protein labeling by Alk-12. To confirm the selective labeling of HIV-1 MA protein that we observed with fluorescence-based click reactions, cell lysates were instead reacted with biotin azide. Biotin-conjugated proteins were precipitated with streptavidin agarose, and bound proteins were released with sodium dithionite, which cleaves a diazo linker within the azidobiotin molecule, enabling selective elution of Alk-4 labeled proteins. Eluents were probed for the MA-Flag proteins, and, in the presence of Alk-4, HIV-1 MA protein was recovered. HIV-1 MA protein recovery was diminished both when a myristoylation-deficient HIV-1 MA protein variant was transfected (MA-G2A) (Fig. 3*B*) and when FASN was inhibited by Fasnall. These results indicate that Alk-4 is metabolized into an FA adduct that is specifically incorporated onto protein myristoylation sites in an FASN-dependent manner, and that can be detected by multiple labeling modalities.

### FASN-dependent and Alk-4–mediated metabolic labeling of endogenous fatty acylated proteins

**Figure 4:**
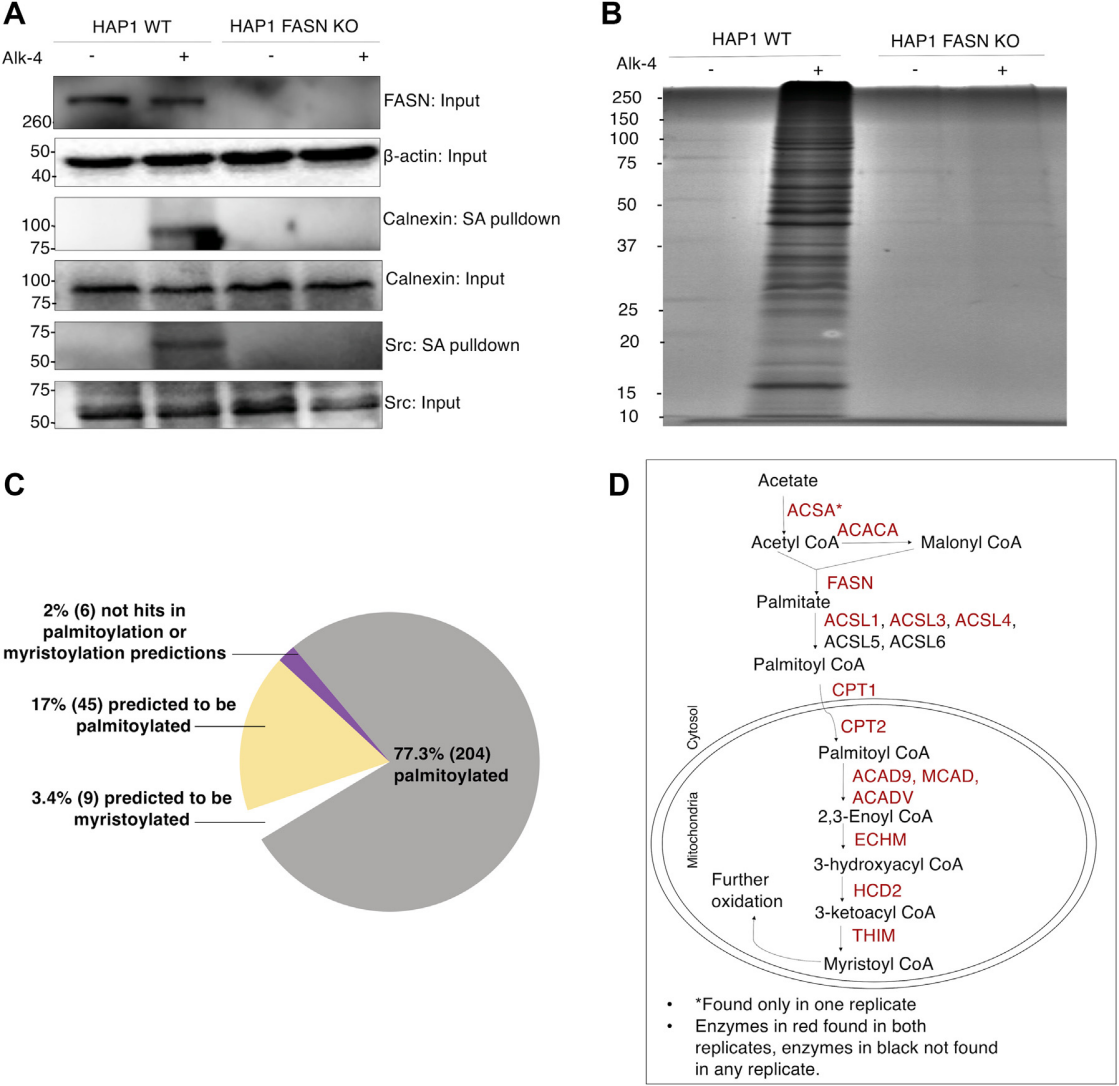
**FASN-dependent purification of myristoylated and palmitoylated proteins from Alk-4 labeled cells.** HAP1 WT and HAP1 FASN KO cells were labeled with Alk-4, click reacted with diazo azidobiotin, and labeled proteins were purified using streptavidin beads and eluted using sodium dithionite. *A*, Western blotting indicates that proteins known to be palmitoylated (calnexin) and myristoylated (Src) can be purified from cells in an Alk-4- and FASN-dependent manner. *B*, Coomassie staining of the streptavidin (SA) pull-down fraction revealed recovery of several proteins only in WT cells labeled with Alk-4. *C*, proteomics analysis of SA pull-down fraction revealed that 77% of proteins selectively recovered in the WT Alk-4 cells are found in at least one palmitoyl proteome or experimentally validated to be palmitoylated based on the SwissPalm database (dataset 3). In addition, 17% of proteins are predicted to be palmitoylated, based both on GPS-lipid analysis using high confidence settings, CSS-Palm, and SwissPalm (dataset 1). GPS-lipid analysis also revealed that 3% of proteins are predicted to be myristoylated (consensus N-terminal glycine and nonconsensus sequence), whereas 2% of the proteins were not hits on prediction algorithms but had isoforms and cysteines as per SwissPalm analysis. *D*, proteomics analysis of SA pull-down fraction (n = 2) revealed recovery of enzymes involved in elongation of short-chain fatty acids as well as enzymes involved in the mitochondrial beta-oxidation pathway for oxidation of long-chain fatty acids. Long-chain fatty acid such as palmitic acid and its oxidized product myristic acid can be used for fatty acylation of proteins.

To test the utility of Alk-4 as a global indicator of FASN-dependent protein acylation, we incubated the human fibroblast-like cell line HAP1 or an FASN-deficient clone of the HAP1 cells with Alk-4 or a vehicle control (dimethyl sulfoxide [DMSO]). Following metabolic labeling of HAP1 cells with Alk-4, cell lysates were reacted with azidobiotin, and labeled proteins were precipitated as described in Figure 3*B*. Eluents were then probed for proteins known to be palmitoylated (calnexin) (37) or myristoylated (Src) (38, 39). In WT HAP1 cells, Alk-4 labeling recovered both calnexin and Src, whereas in FASN-deficient cells, incubation with Alk-4 did not enable calnexin or Src recovery (Fig. 4*A*). To determine the breadth of proteins recovered from cells incubated with Alk-4, we next used mass spectrometry to identify biotinylated proteins from HAP1 cells with or without Alk-4 and with or without FASN. FASN was only recovered from WT HAP1 cells incubated with Alk-4, consistent with the acyl intermediates formed between FASN and the elongating FA chain (40). In total, Alk-4 labeling enabled recovery of 264 proteins in an Alk-4- and FASN-dependent manner (Fig. 4*B*). Of these, 77% (203) have previously been identified in at least one palmitoyl proteome, or they have been experimentally validated to be palmitoylated. These included well-characterized palmitoylation substrates, such as guanine nucleotide-binding protein (G protein), alpha inhibiting 1 (GNA1I) and catenin beta-1 (CTNB1) (Fig. 4*C*, Tables S1 and S3). Of the remaining proteins that were purified, 17% were predicted to be palmitoylated (*e.g.*, SAM domain and HD domain containing protein 1 [SAMHD1]) and 3% were predicted to be myristoylated (*e.g.*, ribosomal protein S6 kinase alpha [KS6A1]) (41, 42) (Fig. 4*C*, Tables S1 and S4). This experiment also recovered, in an Alk-4 dependent manner, several enzymes involved in the metabolism of acetate to palmitate and myristate, including acetyl-CoA synthetase, acetyl-CoA carboxylase 1, and multiple enzymes involved in FA beta-oxidation (Fig. 4*D* and Table S2).

## Discussion

We describe a click chemistry–compatible FASN substrate, Alk-4 (5-hexynoate), which selectively labels both palmitoylated (*e.g.*, IFITM3, CD9) and myristoylated (*e.g.*, HIV-1 MA) proteins. Click chemistry–compatible substrate analogs like Alk-4 overcome several inherent disadvantages of ^3^H radiolabeling, such as long sample processing and film exposure times with low sensitivity (16). While ^14^C radiolabeling might offer comparable or better sensitivity, click chemistry reactions can be easily combined with several detection methods that are compatible with high-throughput applications without the use of radioisotopes, including mass spectrometry–based proteomics, flow cytometry, fluorescence microscopy, and live cell imaging (21). Beyond identification of FASN-dependent protein acylation, Alk-4 has more functionality than a radiolabeled FASN substrate because it can be reacted with azidobiotin to facilitate streptavidin-based purification of FASN-dependent acylated proteins. We are not the first to suggest the utility of Alk-4, which has previously been evaluated as a chemical tool to monitor protein acetylation at shorter time scales, although it was noted that some of the acetylation reporters were incorporated onto proteins by chemical acylation (24). Nevertheless, our finding that FASN activity is required for Alk-4 labeling of multiple proteins, such as IFITM3, HIV-1 MA, calnexin, and Src, strongly supports the use of Alk-4 as a selective reporter of FASN-dependent protein acylation.

FASN activity is required for replication of several enveloped viruses, including chikungunya (10), HIV-1 (9), influenza (43), severe acute respiratory syndrome coronavirus 2 (44), and many others (6, 7, 13). FASN inhibitors, including Fasnall (9, 36) and the TVB compounds (45, 46), have therapeutic potential, and pharmacological inhibition of FASN has been shown to modulate fatty acylation of viral proteins, including chikungunya virus nsP1 palmitoylation (11), severe acute respiratory syndrome coronavirus 2 spike palmitoylation (44), and HIV-1 Gag myristoylation (Fig. 3). In other cases, modulation of FASN activity affects host proteins that regulate infection, including MYD88 palmitoylation (47), and the results presented here that reveal that FASN activity is required for an effective IFNβ immune response against influenza virus, possibly by providing fatty acyl moieties for modification of IFITM3. Increased *de novo* FA biosynthesis and FASN upregulation has also been observed in breast cancer, melanoma, and hepatocellular carcinoma (4), and *de novo* FA biosynthesis and lipogenesis has been shown to be essential for protein palmitoylation of Ras, Wnt (48), calnexin (49), and Src (50) in proliferating cells (51, 52). In tumor cells, FASN inhibition can have consequences beyond inhibition of protein acylation (53); *de novo* FA biosynthesis has also been shown to be essential for membrane remodeling in tumor cells, where palmitate depletion *via* FASN inhibition led to disruption of lipid rafts and signaling pathways, ultimately resulting in apoptosis of tumor cells (54). Thus, *de novo* FA biosynthesis is a broadly utilized fundamental metabolic pathway exploited during carcinogenesis and virus replication, and Alk-4 and its ability to measure flux through the *de novo* FASN pathway provides a new tool to better understand the role of FASN-dependent protein acylation during FASN-dependent pathologies.

## Experimental procedures

### Reagents, transfections, and infections

Reagents, including 5-hexynoic acid (Alk-4), were purchased from Sigma or Thermo Fisher Scientific, unless stated otherwise. Alk-12 and Alk-16 were synthesized by the Hang laboratory according to published protocols (21). Alk compounds were diluted in DMSO. Human embryonic kidney 293T cells were obtained from the American Type Culture Collection and maintained in Dulbecco's modified Eagle's medium supplemented with 10% heat-inactivated fetal bovine serum (FBS; Serum Source International) and 1% penicillin/streptomycin. HAP1 WT and HAP-1 FASN KO cells were obtained from Horizon Discovery and maintained in Iscove's modified Dulbecco's medium supplemented with 10% heat-inactivated FBS and 1% penicillin/streptomycin. HIV-1 MA plasmids pQCXIP-MA-FH (pMA-Flag) and pQCXIP-G2A-FH (pG2A-MA-Flag) were kindly provided by Dr Stephen Goff, Columbia University (55). Hemagglutinin (HA)-tagged IFITM3 and CD9 (22) as well as HA-tagged ZDHHC7 (56) have been described. Cells were transfected using Genefect (Alkali Scientific) or LipoJet transfection reagents (Signagen Laboratories), according to each manufacturer's protocol. Fasnall was synthesized as described (36). Influenza virus H1N1 strain PR8 was propagated in 10-day-old embryonated chicken eggs (Charles River Laboratories) for 48 h at 37 °C as described previously (57, 58). IFNβ-treated HAP1 WT and FASN KO cells were treated with IFN overnight or left untreated as described previously (33) and infected with H1N1 for 24 h (multiplicity of infection = 1). Cells were stained with anti-influenza virus nucleoprotein antibodies to measure percentage of infection by flow cytometry.

### Metabolic labeling, immunoprecipitations, and copper-catalyzed azide–alkyne cycloaddition

Cells were incubated for 24 h with 5 mM Alk-3, 5 mM Alk-4, 50 μM Alk-12, 50 μM Alk-16, or 0.001% DMSO in media supplemented with 1% charcoal-stripped FBS (Serum Source International; catalog number FB02-500CS), and then collected and washed thrice in ice-cold 1× PBS. Cells were lysed for 10 min on ice in 100 μl 1% Brij buffer (1% [w/v] Brij O10, 150 mM NaCl, 50 mM triethanolamine with 1× EDTA-free complete protease inhibitor cocktail). Protein concentration was determined using the bicinchoninic acid assay. Flag precipitations used 500 μg of cell lysate mixed with Protein G-coated agarose beads and incubated with anti-Flag antibody (catalog number: F3165) for 2 h at 4 °C. Anti-HA immunoprecipitations were performed using EZview Red Anti-HA Affinity Gel. Protein-conjugated beads were washed thrice with radioimmunoprecipitation assay buffer (50 mM triethanolamine, 150 mM NaCl, 1% sodium deoxycholate, 1% Triton X-100, and 0.1% SDS). Protein complexes bound to antibody-coated beads were released by adding 4% SDS buffer (150 mM NaCl, 50 mM triethanolamine, and 4% [w/v] SDS), and the click reaction was initiation by addition of 2.75 μl of click chemistry master mix (0.5 μl of 5 mM azidorhodamine or tetramethylrhodamine-5-carbonyl azide (Click Chemistry Tools) in DMSO, 0.5 μl of 50 mM Tris(2-carboxyethyl)phosphine, 0.5 μl of 50 mM CuSO_4_, and 1.5 μl of 2 mM Tris ((1-benzyl-1H-1,2,3-triazol-4-yl)methyl)amine in 1:4 (v/v) DMSO/butanol). Reactions were incubated for 1 h at room temperature, and proteins were eluted from the beads by heating at 95 °C for 5 min in 4× SDS sample loading buffer (40% [v/v] glycerol, 240 mM Tris·chloride, pH 6.8, 8% [w/v] SDS, 0.04% [w/v] bromophenol blue, and 5% 2-mercaptoethanol). Eluted proteins were resolved on 4 to 20% Tris-glycine gels. To detect fluorescently labeled proteins, the gel was destained in 40% distilled water (v/v), 50% (v/v) methanol, 10% (v/v) acetic acid, and visualized using on an Amersham Typhoon 9410 with 532-nm excitation and 580-nm detection filters, or a Biorad Chemidoc MP Imaging System with the rhodamine filters. To remove background fluorescence speckles, the image was despeckled using ImageJ (National Institutes of Health and Laboratory for Optican and Computational Instrumentation, University of Wisconsin). For biotin-based click reactions, Alk-4–incubated cells were lysed with 50 μl 4% SDS buffer with 1× EDTA-free protease inhibitors supplemented with benzonase nuclease (catalog number: E1014). One milligram of cell lysate was resuspended in 445 μl 1× SDS buffer with 1× EDTA-free protease inhibitors and incubated for 1.5 h with 55 μl of click reaction master-mix consisting of 10 μl of 5 mM diazo biotin azide (Click Chemistry Tools), 10 μl of 50 mM Tris(2-carboxyethyl)phosphine, 25 μl of 2 mM Tris((1-benzyl-1H-1,2,3-triazol-4-yl)methyl)amine, and 10 μl of 50 mM CuSO_4_. Proteins were precipitated using chloroform–methanol to remove unreacted biotin azide, and the precipitant was resuspended in 100 μl 4% SDS buffer containing protease inhibitors supplemented and 2 μl of 0.5 M EDTA solution to chelate residual copper. Equivalent amount of protein in 100 μl 4% SDS buffer and 200 μl 1% Brij buffer with EDTA-free protease inhibitors was incubated with 75 μl streptavidin agarose (EMD Millipore) for 2 h at room temperature. Protein-conjugated beads were washed once in PBS/0.2 to 1% SDS, and thrice in PBS. Labeled proteins were selectively eluted by two elutions with 50 mM sodium dithionite, desalted using spin desalting columns, mixed with 4× SDS sample loading buffer, and resolved on 10 to 12% Tris–glycine gel. Coomassie staining was done using standard techniques (59).

### Western blotting

Proteins were transferred onto polyvinylidene fluoride membrane (Bio-Rad), blocked with 5% bovine serum albumin dissolved in 1× Tris-buffered saline containing 0.1% Tween-20. Anti-Flag (catalog number: F3165) and anti-FASN (catalog number: SAB4300700) antibodies were purchased from Sigma and used at a final concentration of 1:1000; anti-calnexin (catalog number: 2679S) and anti-Src (catalog number: 2109S) antibodies were purchased from Cell Signaling Technologies and used at a final concentration of 1:2000. Anti-rabbit secondary antibody (Thermo Fisher Scientific; catalog number: 31460) was used at a final concentration of 1:5000, and antimouse secondary antibody (Cell Signaling Technology; catalog number: 7076S) was used at a final concentration of 1:2000.

### Protein identification

Capillary LC–MS/MS was performed using a Thermo Scientific orbitrap fusion mass spectrometer equipped with an EASY-Spray Sources operated in positive ion mode. Samples were separated on an easy spray nano column (Pepmap RSLC, C18 3 μm 100A, 75 μm × 150 mm; Thermo Scientific) using a 2D RSLC HPLC system from Thermo Scientific. The full scan was performed at Fourier transform mode, and the resolution was set at 120,000. EASY-IC was used for internal mass calibration. Mass spectra were searched using Mascot Daemon by Matrix Science, version 2.3.2, and the database searched against UniProt Human database (version 12032015). Data from two independent experiments were compiled on Scaffold Visualization software (Scaffold 4.9.0; Proteome Software, Inc) (Tables S5 and S6). Proteins were identified based on total spectrum count with a 1% false discovery rate and a minimum of two peptides. Proteins were considered high confidence hits if they had DMSO spectral count of zero and a minimum spectral count of five in both replicates. Putative fatty acylation sites in high confidence proteins were identified in the SwissPalm protein S-palmitoylation database (version 3; https://SwissPalm.org/) using dataset 3 (proteins found in at least one palmitoyl-proteome or experimentally validated to be palmitoylated) and dataset 1 (all datasets). Protein sequences were also searched against GPS-Lipid using high threshold settings (version 1.0; http://lipid.biocuckoo.org/), as described in the Supplementary methods section.

## Data availability

The mass spectrometry proteomics data have been deposited to the ProteomeXchange Consortium *via* the PRIDE (60) partner repository with the dataset identifier (accession number: PXD028244). All other data are included within the article and Supporting information files.

## Supporting information

This article contains supporting information.

## Conflict of interest

The authors declare that they have no conflicts of interest with the contents of this article.

## References

[1] Resh M.D. (2016). Fatty acylation of proteins: The long and the short of it. Prog. Lipid Res..

[2] Suburu J., Gu Z., Chen H., Chen W., Zhang H., Chen Y.Q. (2013). Fatty acid metabolism: Implications for diet, genetic variation, and disease. Food Biosci..

[3] Liu H., Liu J.Y., Wu X., Zhang J.T. (2010). Biochemistry, molecular biology, and pharmacology of fatty acid synthase, an emerging therapeutic target and diagnosis/prognosis marker. Int. J. Biochem. Mol. Biol..

[4] Kuhajda F.P. (2000). Fatty-acid synthase and human cancer: New perspectives on its role in tumor biology. Nutrition.

[5] Zhang H., Li H., Yang Y., Li S., Ren H., Zhang D., Hu H. (2013). Differential regulation of host genes including hepatic fatty acid synthase in HBV-transgenic mice. J. Proteome Res..

[6] Tongluan N., Ramphan S., Wintachai P., Jaresitthikunchai J., Khongwichit S., Wikan N., Rajakam S., Yoksan S., Wongsiriroj N., Roytrakul S., Smith D.R. (2017). Involvement of fatty acid synthase in dengue virus infection. Virol. J..

[7] Li Y., Webster-Cyriaque J., Tomlinson C.C., Yohe M., Kenney S. (2004). Fatty acid synthase expression is induced by the Epstein-Barr virus immediate-early protein BRLF1 and is required for lytic viral gene expression. J. Virol..

[8] Nasheri N., Joyce M., Rouleau Y., Yang P., Yao S., Tyrrell D.L., Pezacki J.P. (2013). Modulation of fatty acid synthase enzyme activity and expression during hepatitis C virus replication. Chem. Biol..

[9] Kulkarni M.M., Ratcliff A.N., Bhat M., Alwarawrah Y., Hughes P., Arcos J., Loiselle D., Torrelles J.B., Funderburg N.T., Haystead T.A., Kwiek J.J. (2017). Cellular fatty acid synthase is required for late stages of HIV-1 replication. Retrovirology.

[10] Bakhache W., Neyret A., McKellar J., Clop C., Bernard E., Weger-Lucarelli J., Briant L. (2019). Fatty acid synthase and stearoyl-CoA desaturase-1 are conserved druggable cofactors of Old World Alphavirus genome replication. Antiviral Res..

[11] Zhang N., Zhao H., Zhang L. (2019). Fatty acid synthase promotes the palmitoylation of Chikungunya virus nsP1. J. Virol..

[12] Krishnan M.N., Ng A., Sukumaran B., Gilfoy F.D., Uchil P.D., Sultana H., Brass A.L., Adametz R., Tsui M., Qian F., Montgomery R.R., Lev S., Mason P.W., Koski R.A., Elledge S.J. (2008). RNA interference screen for human genes associated with West Nile virus infection. Nature.

[13] Martín-Acebes M.A., Blázquez A.B., Jiménez de Oya N., Escribano-Romero E., Saiz J.C. (2011). West Nile virus replication requires fatty acid synthesis but is independent on phosphatidylinositol-4-phosphate lipids. PLoS One.

[14] Hang H.C., Linder M.E. (2011). Exploring protein lipidation with chemical biology. Chem. Rev..

[15] Rodenburg R.N.P., Snijder J., Van De Waterbeemd M., Schouten A., Granneman J., Heck A.J.R., Gros P. (2017). Stochastic palmitoylation of accessible cysteines in membrane proteins revealed by native mass spectrometry. Nat. Commun..

[16] Draper J.M., Smith C.D. (2009). Palmitoyl acyltransferase assays and inhibitors (review). Mol. Membr. Biol..

[17] Gao X., Hannoush R.N. (2018). A decade of click chemistry in protein palmitoylation: Impact on discovery and new biology. Cell Chem. Biol..

[18] Yap M.C., Kostiuk M.A., Martin D.D.O., Perinpanayagam M.A., Hak P.G., Siddam A., Majjigapu J.R., Rajaiah G., Keller B.O., Prescher J.A., Wu P., Bertozzi C.R., Falck J.R., Berthiaume L.G. (2010). Rapid and selective detection of fatty acylated proteins using ω-alkynyl-fatty acids and click chemistry. J. Lipid Res..

[19] Thiele C., Papan C., Hoelper D., Kusserow K., Gaebler A., Schoene M., Piotrowitz K., Lohmann D., Spandl J., Stevanovic A., Shevchenko A., Kuerschner L. (2012). Tracing fatty acid metabolism by click chemistry. ACS Chem. Biol..

[20] Ourailidou M.E., Zwinderman M.R.H., Dekker F.J. (2016). Bioorthogonal metabolic labelling with acyl-CoA reporters: Targeting protein acylation. MedChemComm.

[21] Charron G., Zhang M.M., Yount J.S., Wilson J., Raghavan A.S., Shamir E., Hang H.C. (2009). Robust fluorescent detection of protein fatty-acylation with chemical reporters. J. Am. Chem. Soc..

[22] Yount J.S., Moltedo B., Yang Y.Y., Charron G., Moran T.M., López C.B., Hang H.C. (2010). Palmitoylome profiling reveals S-palmitoylation-dependent antiviral activity of IFITM3. Nat. Chem. Biol..

[23] Chesarino N.M., Hach J.C., Chen J.L., Zaro B.W., Rajaram M.V., Turner J., Schlesinger L.S., Pratt M.R., Hang H.C., Yount J.S. (2014). Chemoproteomics reveals toll-like receptor fatty acylation. BMC Biol..

[24] Yang Y.Y., Ascano J.M., Hang H.C. (2010). Bioorthogonal chemical reporters for monitoring protein acetylation. J. Am. Chem. Soc..

[25] Roth A.F., Wan J., Green W.N., Yates J.R., Davis N.G. (2006). Proteomic identification of palmitoylated proteins. Methods.

[26] Mitchell D.A., Vasudevan A., Linder M.E., Deschenes R.J. (2006). Protein palmitoylation by a family of DHHC protein S-acyltransferases. J. Lipid Res..

[27] Greaves J., Munro K.R., Davidson S.C., Riviere M., Wojno J., Smith T.K., Tomkinson N.C., Chamberlain L.H. (2017). Molecular basis of fatty acid selectivity in the zDHHC family of S-acyltransferases revealed by click chemistry. Proc. Natl. Acad. Sci. U. S. A..

[28] Jennings B.C., Linder M.E. (2012). DHHC protein S-acyltransferases use similar ping-pong kinetic mechanisms but display different acyl-CoA specificities. J. Biol. Chem..

[29] Muszbek L., Haramura G., Cluette-Brown J.E., Van Cott E.M., Laposata M. (1999). The pool of fatty acids covalently bound to platelet proteins by thioester linkages can be altered by exogenously supplied fatty acids. Lipids.

[30] Hallak H., Muszbek L., Laposata M., Belmonte E., Brass L.F., Manning D.R. (1994). Covalent binding of arachidonate to G protein alpha subunits of human platelets. J. Biol. Chem..

[31] Veit M., Reverey H., Schmidt M.F. (1996). Cytoplasmic tail length influences fatty acid selection for acylation of viral glycoproteins. Biochem. J..

[32] Thinon E., Fernandez J.P., Molina H., Hang H.C. (2018). Selective enrichment and direct analysis of protein S-palmitoylation sites. J. Proteome Res..

[33] McMichael T.M., Zhang L., Chemudupati M., Hach J.C., Kenney A.D., Hang H.C., Yount J.S. (2017). The palmitoyltransferase ZDHHC20 enhances interferon-induced transmembrane protein 3 (IFITM3) palmitoylation and antiviral activity. J. Biol. Chem..

[34] Percher A., Ramakrishnan S., Thinon E., Yuan X., Yount J.S., Hang H.C. (2016). Mass-tag labeling reveals site-specific and endogenous levels of protein S-fatty acylation. Proc. Natl. Acad. Sci. U. S. A..

[35] Farazi T.A., Waksman G., Gordon J.I. (2001). The biology and enzymology of protein N-myristoylation. J. Biol. Chem..

[36] Alwarawrah Y., Hughes P., Loiselle D., Carlson D.A., Darr D.B., Jordan J.L., Xiong J., Hunter L.M., Dubois L.G., Thompson J.W., Kulkarni M.M., Ratcliff A.N., Kwiek J.J., Haystead T.A. (2016). Fasnall, a selective FASN inhibitor, shows potent anti-tumor activity in the MMTV-Neu model of HER2(+) breast cancer. Cell Chem. Biol..

[37] Lakkaraju A.K.K., Abrami L., Lemmin T., Blaskovic S., Kunz B., Kihara A., Dal Peraro M., Van Der Goot F.G. (2012). Palmitoylated calnexin is a key component of the ribosome-translocon complex. EMBO J..

[38] Patwardhan P., Resh M.D. (2010). Myristoylation and membrane binding regulate c-Src stability and kinase activity. Mol. Cell. Biol..

[39] Resh M.D. (1994). Myristylation and palmitylation of Src family members: The fats of the matter. Cell.

[40] Wakil S.J. (1989). Fatty acid synthase, a proficient multifunctional enzyme. Biochemistry.

[41] Xie Y., Zheng Y., Li H., Luo X., He Z., Cao S., Shi Y., Zhao Q., Xue Y., Zuo Z., Ren J. (2016). GPS-lipid: A robust tool for the prediction of multiple lipid modification sites. Sci. Rep..

[42] Ren J., Wen L., Gao X., Jin C., Xue Y., Yao X. (2008). CSS-Palm 2.0: An updated software for palmitoylation sites prediction. Protein Eng. Des. Sel..

[43] Munger J., Bennett B.D., Parikh A., Feng X.-J., Mcardle J., Rabitz H.A., Shenk T., Rabinowitz J.D. (2008). Systems-level metabolic flux profiling identifies fatty acid synthesis as a target for antiviral therapy. Nat. Biotechnol..

[44] Lee M., Sugiyama M., Mekhail K., Latreille E., Khosraviani N., Wei K., Lee W.L., Antonescu C., Fairn G.D. (2020). Fatty acid synthase inhibition prevents palmitoylation of SARS-CoV2 spike protein and improves survival of mice infected with murine hepatitis virus. bioRxiv.

[45] de Aquino I.G., Bastos D.C., Cuadra-Zelaya F.J.M., Teixeira I.F., Salo T., Coletta R. Della, Graner E. (2020). Anticancer properties of the fatty acid synthase inhibitor TVB-3166 on oral squamous cell carcinoma cell lines. Arch. Oral Biol..

[46] Zaytseva Y.Y., Rychahou P.G., Le A.T., Scott T.L., Flight R.M., Kim J.T., Harris J., Liu J., Wang C., Morris A.J., Sivakumaran T.A., Fan T., Moseley H., Gao T., Lee E.Y. (2018). Preclinical evaluation of novel fatty acid synthase inhibitors in primary colorectal cancer cells and a patient-derived xenograft model of colorectal cancer. Oncotarget.

[47] Kim Y.-C., Lee S.E., Kim S.K., Jang H.-D., Hwang I., Jin S., Hong E.-B., Jang K.-S., Kim H.-S. (2019). Toll-like receptor mediated inflammation requires FASN-dependent MYD88 palmitoylation. Nat. Chem. Biol..

[48] Kaemmerer E., Gassler N. (2016). Wnt lipidation and modifiers in intestinal carcinogenesis and cancer. Cancers (Basel).

[49] Lynes E.M., Raturi A., Shenkman M., Sandoval C.O., Yap M.C., Wu J., Janowicz A., Myhill N., Benson M.D., Campbell R.E., Berthiaume L.G., Lederkremer G.Z., Simmen T. (2013). Palmitoylation is the switch that assigns calnexin to quality control or ER Ca2+ signaling. J. Cell Sci..

[50] Kim S., Alsaidan O.A., Goodwin O., Li Q., Sulejmani E., Han Z., Bai A., Albers T., Beharry Z., Zheng Y.G., Norris J.S., Szulc Z.M., Bielawska A., Lebedyeva I., Pegan S.D. (2017). Blocking myristoylation of Src inhibits its kinase activity and suppresses prostate cancer progression. Cancer Res..

[51] Fiorentino M., Zadra G., Palescandolo E., Fedele G., Bailey D., Fiore C., Nguyen P.L., Migita T., Zamponi R., Di Vizio D., Priolo C., Sharma C., Xie W., Hemler M.E., Mucci L. (2008). Overexpression of fatty acid synthase is associated with palmitoylation of Wnt1 and cytoplasmic stabilization of beta-catenin in prostate cancer. Lab. Invest..

[52] Yao C.H., Fowle-Grider R., Mahieu N.G., Liu G.Y., Chen Y.J., Wang R., Singh M., Potter G.S., Gross R.W., Schaefer J., Johnson S.L., Patti G.J. (2016). Exogenous fatty acids are the preferred source of membrane lipids in proliferating fibroblasts. Cell Chem. Biol..

[53] Chen M., Huang J. (2019). The expanded role of fatty acid metabolism in cancer: New aspects and targets. Precis. Clin. Med..

[54] Ventura R., Mordec K., Waszczuk J., Wang Z., Lai J., Fridlib M., Buckley D., Kemble G., Heuer T.S. (2015). Inhibition of de novo palmitate synthesis by fatty acid synthase induces apoptosis in tumor cells by remodeling cell membranes, inhibiting signaling pathways, and reprogramming gene expression. EBioMedicine.

[55] Zhu Y., Luo S., Sabo Y., Wang C., Tong L., Goff S.P. (2017). Heme oxygenase 2 binds myristate to regulate retrovirus assembly and TLR4 signaling. Cell Host Microbe.

[56] Hach J.C., McMichael T., Chesarino N.M., Yount J.S. (2013). Palmitoylation on conserved and nonconserved cysteines of murine IFITM1 regulates its stability and anti-influenza A virus activity. J. Virol..

[57] Yount J.S., Kraus T.A., Horvath C.M., Moran T.M., López C.B. (2006). A novel role for viral-defective interfering particles in enhancing dendritic cell maturation. J. Immunol..

[58] Moltedo B., Li W., Yount J.S., Moran T.M. (2011). Unique type I interferon responses determine the functional fate of migratory lung dendritic cells during influenza virus infection. PLoS Pathog..

[59] Merril C.R. (1990). Gel-staining techniques. Methods Enzymol..

[60] Perez-Riverol Y., Csordas A., Bai J., Bernal-Llinares M., Hewapathirana S., Kundu D.J., Inuganti A., Griss J., Mayer G., Eisenacher M., Pérez E., Uszkoreit J., Pfeuffer J., Sachsenberg T., Yilmaz S. (2019). The PRIDE database and related tools and resources in 2019: Improving support for quantification data. Nucleic Acids Res..

